# Periodontal Health Status in Adults Exposed to Tobacco Heating System Aerosol and Cigarette Smoke vs. Non-Smokers: A Cross-Sectional Study

**DOI:** 10.3390/dj12020026

**Published:** 2024-01-29

**Authors:** Ivana Mišković, Davor Kuiš, Stjepan Špalj, Aleksandar Pupovac, Jelena Prpić

**Affiliations:** 1 Clinical Hospital Centre Rijeka, Krešimirova 40, 51000 Rijeka, Croatia; ivana.sciran@fdmri.uniri.hr (I.M.); davor.kuis@uniri.hr (D.K.); stjepan.spalj@fdmri.uniri.hr (S.Š.); 2 Department of Periodontology, Faculty of Dental Medicine, University of Rijeka, Krešimirova 40-42, 51000 Rijeka, Croatia; 3 Department of Dental Medicine, Faculty of Dental Medicine and Health, Josip Juraj Strossmayer University of Osijek, Crkvena 21, 31000 Osijek, Croatia; 4 Department of Orthodontics, Faculty of Dental Medicine, University of Rijeka, Krešimirova 40-42, 51000 Rijeka, Croatia

**Keywords:** cigarette smoking, electronic nicotine delivery system, periodontics, periodontitis, tobacco, smokers

## Abstract

Tobacco heating systems (THS) are new products on the market, advertised as a less harmful alternative for smokers, in which tobacco is heated and not burned like in conventional cigarettes. This research explored the effect on periodontal tissues in contact with heating and burning tobacco residual products (smoke and tobacco). Methods: The sample included 66 subjects, patients of the Clinic of Dentistry in Rijeka, Croatia, aged 26–56 (median 38), 64% females. Three age- and gender-matched groups were formed (each *N* = 22): non-smokers, classic cigarettes smokers and THS smokers. Probing depth (PD) and clinical attachment loss (CAL) were primary research parameters. Results: Three groups differed in average PD and CAL (*p* ≤ 0.002), with cigarette smokers having the highest and non-smokers the lowest values (*p* ≤ 0.002). THS consumers generally had lower values of periodontal indices than smokers, but only CAL differed significantly (*p* = 0.011). Periodontal indices CAL and PD were worse in THS consumers than non-smokers, but they did not reach a level of statistical significance. Cigarette smoking was the only predictor of periodontitis (average CAL ≥ 4 mm) in logistic regression models, with an odds ratio of 4.7 (95% confidence interval 1.2–18.3; *p* = 0.027). Conclusions: Exposure to nicotine-containing aerosol of THS in adults has a less harmful effect on periodontal tissues, measurable through periodontal indices (PD and CAL), compared to burning tobacco of conventional cigarettes. THS, presented as an alternative product to classic cigarettes, also has a detrimental effect on the periodontium.

## 1. Introduction

The link between smoking and periodontitis has been well known for several decades, and a large number of studies demonstrated beyond doubt that cigarette smoke has a marked negative effect on periodontal tissues. The fact that a person is a smoker increases the odds ratio of acquiring periodontal disease by 85% [1].

Tobacco smoke as a product of conventional cigarettes contains a wide variety of harmful, mutagenic and carcinogenic chemicals such as nicotine, carbon monoxide, arsenic, hydrogen cyanide, benzene, reactive radicals, and tobacco-derived nitrosamines [2]. The effects of these cigarette smoke chemicals on the hard and soft tissues of the oral cavity are very well known and documented. Cancer of the oral cavity and pharynx, periodontitis, dental caries, oral pain, and diminished salivary flow are just a few of many conditions found in smokers. Cigarette smoke significantly affects the immune and circulatory systems, influencing the incidence and progression of periodontal disease through various pathways [2,3].

Smokers typically exhibit deeper periodontal probing depths, greater clinical attachment loss, gingival recession, alveolar bone loss, and tooth loss. Gingival bleeding is suppressed in smokers; this fact is often misleading since it may suggest the absence of inflammation, but the opposite is true. It has been proved that smoking affects the vasomotility of blood vessels, especially vasoconstriction in heavy and long-term, ‘chronic’, tobacco smokers, interferes with angiogenesis, and reduces the number of blood vessels [4,5,6]. Bleeding of the gingiva is dose dependent, and the effect of smoking reaches a plateau at 10 to 20 cigarettes per day [5]. Disturbed and reduced gingival microcirculation leads to suppressed perfusion and reduced healing potential, poorer response to periodontal therapy, and finally, worse prognosis [4,5,6].

Many of these changes result from toxins produced during tobacco pyrolysis. Attempts to mitigate these effects led to the development of alternatives like e-cigarettes and the tobacco heating system (THS), with IQOS^®^ (Phillip Morris International, Inc., (PMI), Stamford, Connecticut) being a novel hybrid product [7]. IQOS^®^, as a kind of tobacco heating system, is a novel hybrid product on the market, a combination of conventional cigarettes and electronic ones, powered by a rechargeable battery. It consists of three main components: a battery-powered tobacco heating holder, a tobacco stick (called a HeatStick or HEETS), and a charger. A disposable tobacco stick is inserted into a slot and then heated [2].

IQOS^®^ claims to be a less harmful alternative to conventional cigarettes. Heating tobacco instead of burning it releases fewer harmful ingredients according to the U.S. Food and Drug Administration. Tobacco in IQOS^®^ is heated up to 350 °C but it does not burn like in classic cigarettes, and does not produce fire, ash, or smoke. By heating the tobacco in a THS cigarette, nicotine, volatile substances, and glycerol are released, creating an aerosol which is mainly a product of evaporation and distillation, not combustion and pyrolysis as in conventional cigarettes [3,8,9,10]. It satisfies nicotine cravings while allegedly avoiding adverse health consequences. In vitro studies suggest that IQOS^®^ is less toxic to oral cells compared to traditional cigarettes. Cigarette smoke reduces the viability, proliferation, and migration of oral cells, reduces the production of inflammatory mediators, but also stops cell cycle and initiates apoptosis. In contrast, IQOS^®^ seems to be less toxic for oral fibroblasts and keratinocytes and it is associated with reduced human keratinocyte apoptosis [11]. According to Yoshioka, the use of heated tobacco products was associated with the prevalence of self-reported periodontal disease in comparison with never-users. But former users, classic cigarette smokers, and combined users showed the same kind of association as THS users. Potentially harmful substances in somewhat higher concentrations present in heated aerosol could influence a higher prevalence of periodontal disease in that group of tobacco smokers. Due to the above facts, it was assumed that THS aerosol may be harmful for the oral cavity like classic cigarette smoke [12]. In vitro wound-healing evaluation was made after dental implantation on a animal model (L929 mouse fibroblast cell) when exposed to 2.5 and 5% cigarette smoke extract from THS aerosol and classic cigarette smoke. It was concluded that THS even in lower concentrations of 2.5% can be more toxic than combustive cigarette smoke, so it is considered as a risk factor which could compromise wound healing of dental implants [13]. According to one study, e-cigarettes and THS products have a minor influence on oxidative stress, platelet activation, and blood pressure when compared to traditional cigarettes [14].

It may be observed that the research on THS users indicates potential negative effects on periodontal and peri-implant mucosa [15]. The impact of THS aerosol on oral and periodontal tissues is underexplored, with limited clinical and in vitro studies.

Therefore, the aim of this study was to assess the association between THS consumption and periodontal parameters, expecting differences compared to conventional cigarette smokers and non-smokers. The null hypothesis of the study was that periodontal indices were lower in non-smokers (implying better periodontal status) than THS consumers, and lower in THS than cigarette smokers. Reduced periodontal probing depths, gingival recessions and tooth mobility, lower plaque accumulation, less attachment loss and fewer furcation defects, but a greater extent of gingivitis will be expected in THS users compared to classic cigarette smokers. Furthermore, worse periodontal status in THS and cigarette smokers is presumed compared to non-smokers.

## 2. Materials and Methods

### 2.1. Study Participants and Selection Criteria

The design of this study was an observational, cross-sectional study with sample stratification according to tobacco exposure. A convenience sample consisted of 66 consecutive patients of the Clinic of Dentistry, Clinical Hospital Center Rijeka, Croatia, who came for an examination at one of the six departments. Three groups were formed (each N = 22): (I) subjects smoking classic cigarettes, (II) users of tobacco heating devices (THS), i.e., IQOS^®^ smokeless cigarette consumers, and (III) subjects who have never smoked either classic cigarettes or used the THS system, i.e., have non-smoking status. Firstly, the group of IQOS^®^ users was formed, since they were least numerous, and then matched by age and gender with non-smokers and cigarette smokers. The subjects were recruited between 1 June 2022 and 1 June 2023.

A sample size was calculated based on previous research [16]. If probing depth is expected to be 4.5 mm in smokers, 4.1 in THS, and 4.5 in non-smokers with a standard deviation of 0.4 in each group, taking into account the Bonferroni correction for multiple comparisons and drop-outs, 22 subjects are needed in each group to detect differences among groups. A calculation was made with a test power of 0.8 and a significance level of 0.05 using an online sample size calculator v. 1.061 (University of Vienna, Vienna, Austria) [17].

The research question, formulated using the PICO strategy, aimed to investigate the impact of aerosol from tobacco heating systems compared to tobacco cigarette smoke on periodontal tissues in adult smokers [18].

P (Population): Adult smokers of classic cigarettes and THS users;
I (Intervention): Aerosol from tobacco heating system and tobacco cigarette smoke;
C (Comparison): Non-smokers;
O (Outcome): Clinical periodontal tissue parameters.

The clinical question in “PICO” format was: Is there a significant difference in clinical parameters of periodontal tissues in tobacco heating system users when compared to classic cigarettes users and non-smoking subjects?

Inclusion criteria were good general health, absence of any systemic, metabolic (diabetes), cardiovascular or any other infectious or inflammatory diseases other than periodontitis, absence of any lesions below, at or above the level of the oral mucosa, and a minimum of 20 healthy teeth. Smokers had to fulfill the criteria for smoking experience which was at least 3 years of smoking (classic cigarettes or IQOS^®^) and daily consumption which should not be less than 5 cigarettes or heat sticks per day.

Selected patients were only cigarette smokers for group I, and the same rule of selection was valid for THS users for group II.

The exclusion criteria were modifiers of the supragingival and/or subgingival microbiological profile which can affect the accuracy of the measured periodontal indices [4]. Minors, pregnant women, subjects who use oral probiotics, subjects who had been treated with antibiotic therapy within the last six months, subjects who regularly use oral chlorhexidine-based antiseptics, and subjects under immunosuppressive therapy or any medication therapy which may affect periodontal health have been excluded from the research and as well as those who had previously undergone periodontal therapy.

### 2.2. Questionnaire 

Oral hygiene habits, smoking experience and daily consumption of tobacco sticks or cigarettes were assessed by a questionnaire consisting of 7 questions. A single investigator (IM) used a questionnaire to collect relevant information. Questions had one or more possible answers (Appendix A).

### 2.3. Clinical Examination

Clinical examination included examination of all teeth except third molars. A millimeter graduated PCP-15 UNC periodontal probe (Hu-Friedy, Chicago, IL, USA) was used to record the following periodontal indices: probing depth (PD), Full Mouth Bleeding Score (FMBS), Full Mouth Plaque Score (FMPS), gingival recession (GR), tooth mobility (TM), furcation defects (FD), and clinical attachment level (CAL). PD and GR measurements were measured at 6 sites per tooth (mesio-buccal, middle of the buccal surface, disto-buccal, disto-oral, middle of the oral surface, mesio-oral).

PD is the distance from the cementum–enamel junction (CEJ) to the bottom of the periodontal pocket (in millimeters). PD suggests a presence of periodontal disease if it reaches the value of ≥3 mm [19].

FMBS is the number of tooth sites where bleeding is recorded, divided by total number of available sites multiplied by 100 (expressed as a percentage). The presence or absence of bleeding was recorded 30 s after measuring the pocket depth. FMBS is an indicator of gingivitis [19]. Gingivitis in this study applies to inflammatory conditions induced by plaque accumulation on the tooth surface. It is characterized by gingival redness and edema and the absence of periodontal attachment loss [20].

FMPS is the number of tooth sites where plaque is recorded, divided by total number of available sites multiplied by 100 (expressed as a percentage). A periodontal probe is pulled along the line of gingival margin and the presence of plaque is noted [19].

GR is the distance from CEJ to the free gingival margin or to the margin of the cervical restoration (expressed in millimeters) [19].

TM is graded by applying pressure to the tooth with two handles of metal dental instruments while moving it in buccolingual direction. It is classified by Miller scale of tooth mobility: 0, 1, 2, or 3. A score of 0 implies absence of tooth mobility or physiologic mobility; score 1 is an indicator of mobility greater than physiologic; score 2 is given when mobility is <1 mm in horizontal direction; and score 3 is given when buccolingual mobility is >1 mm, associated with its axial movement [21].

FD is the amount of hard tissue destruction in a multi-rooted tooth. A classification system by Hamp, Nyman, and Lindhe, referring to horizontal attachment loss, was used: 1: horizontal attachment loss < 3 mm of the total width of the furcation area; 2: horizontal attachment loss > 3 mm; and 3: destruction of the periodontal tissue encompassing the total width of the furcation area [22].

CAL is the distance from CEJ to the bottom of the gingival sulci or periodontal pocket. It is expressed in millimeters and suggests the amount of hard and soft tissue loss around teeth [19].

The participants were briefed thoroughly about the study and their voluntary participation, and they all signed written consents.

### 2.4. Statistical Analysis

Frequencies among groups were compared by χ^2^ and Fisher exact tests. The Z-test for proportions with Bonferroni correction was used for multiple comparisons after χ^2^ test. Since there were less than 30 participants per group and continuous variables did not have normal distribution (checked by Shapiro–Wilk’s test), non-parametric statistics was used, and central tendency with dispersion was presented with medians and interquartile range. Continuous variables were analyzed using the Kruskal–Wallis and Mann–Whitney tests with Bonferroni correction of *p* value for multiple comparisons. Effect size for χ^2^ and Fisher tests was quantified by Cramer’s V, by formula ε^2^ = H/[(n^2^ − 1)/(n + 1)] for Kruskall–Wallis, and by the formula r = Z/√N for Mann–Whitney test. The Cohen criteria were used for interpretation: r = 0.25–0.3 = small effect size, 0.3–0.5 = moderate, 0.5–0.7 = large, and >0.7 = very large. For interpretation of Cramer’s V, the same criteria were used, while for ε^2^, squared values of r were used. Logistic regression was used to analyze predictors of periodontitis. Commercial software was used (SPSS IBM 22.0, IBM Corp., Armonk, NY, USA).

### 2.5. Ethical Considerations

This study followed the Declaration of Helsinki guidelines regarding medical protocol and ethics, and the protocol was approved by the Ethics and Research Committee of Faculty of Dental Medicine University of Rijeka on 7 April 2022 (2170-137-006-01-23-60) and Clinical Hospital Center Rijeka, Croatia on 6 March 2022. (2170-29-02/1-23-2).

## 3. Results

The subjects were 26–56 years old (median 38; interquartile range 34–54), and 42/66 (64%) were female. There were no major differences in oral hygiene protocols or teeth brushing frequency among the three groups. All participants used a toothbrush for oral hygiene, 23/66 (35%) used floss, 20/66 (30%) used an interdental brush, and 17/66 (26%) used mouthwash. Participants mostly brushed their teeth twice a day (48/66 (73%)) or ≥3× (12/66 (18%)).

The cigarette smokers had a longer smoking experience than IQOS^®^ users in general, with a moderate effect size (≥5 years 91% vs. 47%; *p* = 0.034; V = 0.367). To control the effect of smoking experience, 13 IQOS^®^ users with smoking experience ≥ 5 years were matched by age, gender, and duration of smoking status with 13 cigarette users and compared. PD was higher in cigarette smokers than IQOS^®^ users, with a moderate effect size (median 3.3 vs. 2.5 mm; *p* = 0.042; r = −0.397; Table 1). Other periodontal indices did not differ significantly, although CAL was greater in smokers than IQOS users (median 3.4 vs. 2.8 mm).

Daily usage of cigarettes and heat sticks was similar (≥10 per day 18/22 (53%) in IQOS^®^ group and 16/22 (47%) in cigarette group).

**Figure 1:**
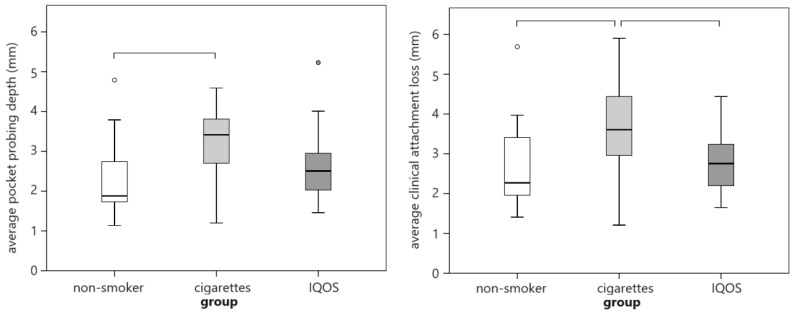
Comparison of average probing depth and clinical attachment loss between nicotine exposure groups. Circles represent outliers, while horizontal lines connect groups that differ significantly.

The three groups differed only in average CAL and PD (*p* ≤ 0.002), with a moderate effect size (ε^2^ = 0.214 for PD and 0.185 for CAL). Cigarette smokers had the highest and non-smokers the lowest values, with a large effect size for PPD and moderate for CAL (r = 0.524 and 0.467; *p* ≤ 0.002; Table 1 and Figure 1. IQOS^®^ consumers had lower values than smokers, but these were significant only for CAL, with a moderate effect size (r = 0.383; *p* = 0.011). The IQOS^®^ group had worse periodontal conditions (CAL and PD) than the non-smoker group, but it did not reach a level of statistical significance (Table 2).

**Figure 2:**
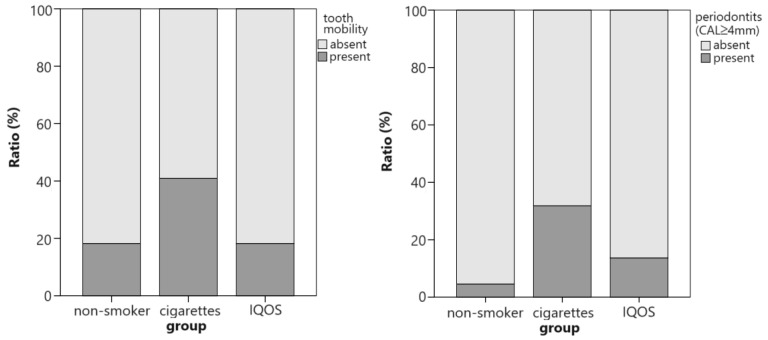
Prevalence of teeth with mobility and periodontitis among groups. Differences between the cigarette and IQOS^®^ groups were not significant.

When periodontitis was defined as having an average CAL ≥ 4 mm, its prevalence differed among groups, with a moderate effect size (*p* = 0.047; V = 0.304) [23]. Average CAL ≥ 4 mm was more prevalent in the cigarette smokers group (7/22; 32%) than the IQOS^®^ users (3/22; 14%) and non-smokers (1/22; 5%). The difference between the last two groups was not significant. The groups did not differ significantly in terms of tooth mobility, but classic cigarette smokers had the highest prevalence of mobility, while non-smokers and IQOS^®^ smokers had the same prevalence (Figure 2).

**Figure 3:**
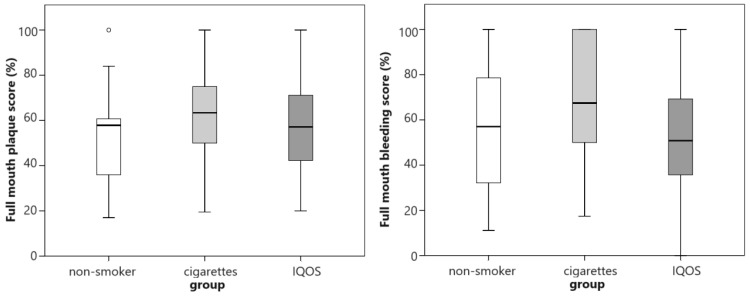
Extent of plaque accumulation and the extent of gingivitis between groups. Circles represent outliers.

The extent of plaque accumulation and gingivitis was highest in smokers and lowest in IQOS^®^ users, but the differences were not statistically significant (Figure 3).

**Figure 4:**
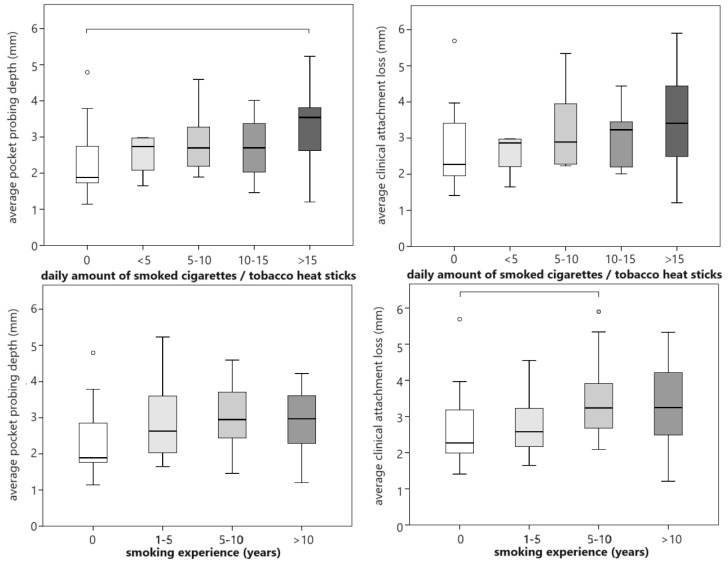
Relationship between daily nicotine exposure and smoking experience with probing depth and attachment loss. Circles represent outliers, while horizontal lines connect groups that differ significantly.

The PD significantly differed between the categories of daily nicotine exposure (*p* = 0.011), but only significantly between non-smokers and those that smoked ≥15 cigarettes/sticks per day (*p* = 0.004; r = 0.521; Figure 4). The CAL and PD differed between categories of smoking experience (*p* ≤ 0.030), but multiple comparisons only detected a difference in CAL between non-smokers and those that had smoked for 5–10 years (*p* = 0.045; r = 0.418; Figure 4). When the categories were dichotomized (smoking experience ≥ 5 years and daily nicotine consumption ≥ 10 cigarettes/heat sticks), significant differences among the groups were detected (*p* ≤ 0.012; r = 0.309–0.503), with worse conditions when smoking experience was ≥5 years and daily nicotine consumption was ≥10 cigarettes/heat sticks. No significant relationships were detected among smoking experience, daily nicotine consumption, and other periodontal indices.

In univariate analysis (Fischer exact test), periodontitis was associated with classic cigarette smoking (*p* = 0.033; V = 0.287; OR 4.7; 95% CI 1.2–18.3) and daily nicotine consumption (dichotomized with limit ≥ 10 cigarettes/IQOS^®^ heat sticks) (*p* = 0.045; V = 0.271; OR 5.4; 95% CI 1.1–27.3). It was not related to gender, age (dichotomized with limit ≥ 41 years), smoking in general, smoking IQOS^®^, smoking experience (dichotomized with limit ≥ 5 years), use of interdental brush, floss or mouthwash, or the frequency of daily brushing. When IQOS^®^ users with smoking experience of up to 5 years (N = 9) and more than 5 years (N = 13) were compared according to PD and CAL as a marker of periodontitis, there was also no statistical significance. Also, there were no differences or statistical significances in other parameters (FMPS, FMBS, TM, GR, FD).

In multiple logistic regression, the following parameters were tested: gender (0 = F; 1 = M), age (0 ≤40; 1 ≥41 years), smoking IQOS^®^ (0 = no; 1 = yes), classic cigarette smoking (0 = no; 1 = yes), daily nicotine consumption (0 ≤9 cigarettes/IQOS^®^ heat sticks; 1 ≥10), smoking experience (0 ≤4 years; 1 ≥5 years), frequency of daily brushing (0 ≤2×; 1 ≥3×), interdental brush use (0 = no; 1 = yes), floss use (0 = no; 1 = yes), and mouthwash use (0 = no; 1 = yes). Several approaches were used (stepwise hierarchical, forward, and backward). The model that included significant variables from univariate analyses (cigarette smoking and daily nicotine consumption) was not significant (Model 1; Table 3). The stepwise forward approach demonstrated that the best model was the one that had only cigarette smoking as the predictor of periodontitis (average attachment loss ≥ 4 mm), with an odds ratio of 4.7 (95% confidence interval 1.2–18.3; *p* = 0.027). The model correctly classified 83% of the cases with Negelkerke’s pseudo R^2^ = 0.126 (Model 2, Table 3).

## 4. Discussion

This research demonstrated that aerosol from the novel tobacco heating system, IQOS^®^, has a detrimental effect on periodontal tissues, albeit significantly lower than the burned cigarette smoke from conventional cigarettes. IQOS^®^ consumers in this study had less overall smoking experience compared to cigarette smokers, possibly contributing to a better periodontal status. The fact that the launch of IQOS^®^ in Croatia was in December 2017 must be taken into consideration [24].

IQOS^®^ consumers resembled non-smokers more than cigarette smokers in terms of periodontal indices. However, the nicotine-containing aerosol from THS proved to be not entirely harmless to the periodontium. As for the available scientific evidence, no literature comparing periodontal status among cigarette smokers, THS smokers, and non-smokers was found. One study on smokers switching to IQOS^®^ demonstrated favorable changes in periodontal disease parameters [25]. The fact that cigarette smokers had a longer smoking experience compared to the IQOS users, may create a bias since the harmful effect of tobacco use is time and dose dependent; the duration of smoking was longer in the cigarette group, and this fact may have accounted for more severe findings in periodontal parameters. This problem was addressed by multiple logistic regression analyses controlling the effect of all parameters on periodontitis.

Cigarette smokers had the highest values of probing depth (PD), gingival recession, plaque and bleeding scores, and clinical attachment loss (CAL); tooth loss was comparable in the cigarette and IQOS^®^ groups. Statistical significance was limited, however, to PD and CAL which are indicative of active periodontal disease. The association between smoking and periodontal disease was confirmed, with smoking relating to a 4.7 times higher odds ratio for periodontitis. Daily consumption, not duration, was linked to periodontitis. As for the finding that values of gingival recession (GR) were lower in IQOS^®^ smokers (without reaching the level of statistical significance), and no differences were demonstrated between cigarette smokers and non-smokers, we could speculate that it only shows that gingival recession may not exclusively be attributed to smoking, but other factors (such as tooth brushing technique, orthodontic factors, and occlusal trauma) should also be considered. 

Surprising results in this investigation were the values for FMBS: this investigation showed that bleeding of the gums was most prevalent in cigarette smokers, and least in IQOS^®^ smokers, despite the duration of smoking history. These differences could only be attributed to some patient-related factors, such as previous instructions for oral hygiene maintenance and subsequent plaque levels.

Concerning IQOS^®^, while it may expose users to lower levels of certain toxins compared to classic cigarettes, it could expose them to higher levels of unrecognized harmful toxins [9,25]. In 2018, WHO released the first ‘Heated tobacco products information sheet’ to provide information on tobacco heating systems or heated tobacco products, and in 2020, the second edition was published. According to these documents, all kinds of tobacco products are harmful, including heated tobacco products. THS contains significantly higher dosages of 20 harmful and potentially harmful chemicals, some of them carcinogens, when compared to cigarette smoke [26]. The European Respiratory Society (ERS) also gave their opinion regarding tobacco heating products in which they concluded that this kind of product is also addictive, carcinogenic, and damaging to the lungs and human health like classic cigarettes [27].

Based on our results, it is hard to claim beyond doubt that heated tobacco is less harmful. Nowadays, tobacco heating system products like IQOS^®^ are promoted as products causing reduced harm, and in some countries, they are sold without graphic warning labels [28]. They are new, modern, and tempting alternatives to tobacco and are likely to be used by teenagers and young adults and by those who have never smoked before. Young adults are a target group for this kind of tobacco industry, because they are marked as the ones with the most substantial economic prospects. Also, people accept consumption of THS product more easily due to its absence of tobacco smoke [29]. It is important to emphasize that tobacco heating systems have a harmful effect and have consequences on the periodontium when compared to non-smokers, as our study presented. Theoretically, the absence of smoke and tar may be beneficial compared to tobacco smoke. In addition to that, we demonstrated that the IQOS^®^ group had lower values of the measured periodontal parameters.

However, whether it was better for gingival tissues or lungs is beyond the scope of the current observational study.

There are some concerns and limitations of our study. The study was observational, so unmeasured confounders may be present. Furthermore, this is a cross-sectional study, so it could not refer to longitudinal causality and interconnection.

One of this observational study’s limitations is its small sample size of 66 subjects, requiring future larger-scale investigations with a diverse population. Although the sample size was calculated, it was evidently underestimated and was not sufficient to detect differences in all parameters. Furthermore, the study population’s relatively poorer oral health challenges generalizability. Another disadvantage of the study is the use of a convenient sample. Patients who come to the University Dental Clinic are relatively sicker in terms of oral health, so it is questionable whether the findings can be applied to the general population. Patients were enrolled from six different dental departments, including those who came only for consultations for esthetic dental problems. An advantage of the study is the age and gender matching of the groups. Further studies that can avoid these limitations are needed.

The public health impact of THS products depends not only on whether they are less harmful than traditional cigarettes, but whether they encourage an increase or decrease in the prevalence of smoking. Findings from selected studies suggest that heat-not-burn tobacco products may create new nicotine-addicted populations [29].

IQOS^®^ may be marketed as a safer alternative to combustive cigarettes, but it is essential and crucial for its users to be aware of potential and actual health risks and consequences for the oral cavity, as we demonstrated in our study. It is important to emphasize that smoking THS products has harmful effects when compared to non-smoking. THS users need to prioritize their oral health by seeking regular dental check-ups, good oral hygiene maintenance, and considering smoking cessation programs. With regular dental check-ups, all potential findings on oral tissues can be recognized in early phases so the potential of treatment success rises and harmful tissue effects can be minimized. Young non-smokers, IQOS^®^ users, or cigarette smokers should be properly informed and educated about the benefits of non-smoking by general health or dental practitioners because they can be easily misled by well-thought-out marketing of the tobacco industry. Reduced risks of periodontal disease, enhanced healing and recovery of the periodontium, and improved esthetics of the teeth with regard to discoloration of teeth, gums, and bad breath are all key arguments to encourage non-smokers to remain non-smokers and smokers to abandon their bad habit [4,30].

The results of this research can help in understanding the effects of smokeless, heated tobacco systems on periodontal tissues and oral health. Also, these systems can help dentists and physicians in counseling patients about the harmful effects of smoking, regardless of whether it is burned or heated.

Smoking and periodontal disease are important public health issues, and by reducing the number of smokers, the number of potential periodontitis patients decreases too, which may lead to further reductions in economic burdens and periodontitis-associated systemic illnesses like diabetes [31,32,33,34].

The hereby presented findings are useful to public health experts and competent authorities who can form guidelines and recommendations for health professionals as well as the general population.

While no tobacco product can be considered safe and risk free, future research should explore short- and long-term effects on oral and general health in longitudinal studies, targeting a broader population, matching smoking experience and daily consumption, and analyzing the oral microbiome [35]. Also, future investigations could focus on the use of adjuvants in home oral care (like chlorhexidine, ozone/based gels, etc.) of cigarette smokers or THS users and compare its effect on periodontal tissues after periodontal therapy in both groups [36,37].

## 5. Conclusions

The presented results may support the hypothesis that exposure to the nicotine-containing aerosol of heated tobacco products in adults is less harmful to periodontal tissues compared to burning tobacco in conventional cigarettes. THS, presented as an alternative product to classic cigarettes, also has detrimental effects on the periodontium.

## Figures and Tables

**Table 1:** Comparison of sociodemographic, smoking, and oral hygiene variables among groups.

	Non-Smokers	IQOS	Cigarette Smokers	*p*
Age ^b^	38.5 (33.0–44.5)	37.0 (33.8–44.3)	38.0 (34.0–44.8)	0.998 ^a^
sex (proportion of females)	14/22	14/22	14/22	1.000 ^c^
smoking experience (≥5 years)	-	13/22	20/22	0.034 ^d^
daily nicotine consumption (≥10 cigarettes/heat sticks)	-	18/22	16/22	0.721 ^d^
frequency of daily brushing (≥3×)	4/22	5/22	3/22	0.737 ^c^
floss use	7/22	10/22	6/22	0.420 ^c^
interdental brush use	7/22	9/22	4/22	0.256 ^c^
mouthwash use	5/22	6/22	6/22	0.924 ^c^

^a^ Kruskall–Wallis test. ^b^ Medians with interquartile range (IQR) in parentheses, ^c^ χ^2^ test, ^d^ Fisher exact test.

**Table 2:** Periodontal disease indices in relation to smoking status.

	Non-Smokers	IQOS	Cigarette Smokers	*p* ^a^
No. of MT ^b,c^	1 (0–3)	2 (0–3)	2 (0–7)	0.211
PD	1.88 (1.72–2.80)	2.51 (2–3.06)	3.42 (2.66–3.82)	0.001
GR	0.31 (0.01–0.46)	0.24 (0.1–0.47)	0.34 (0.11–0.82)	0.408
FD	0 (0–0)	0 (0–0)	0 (0–0)	0.751
TM	0 (0–0)	0 (0–0)	0 (0–1.03)	0.266
FMPS	57.85 (35.79–62.49)	57.14 (41.54–72.11)	63.39 (49.04–76.78)	0.408
FMBS	57 (31.10–82.96)	50.78 (35.43–69.92)	67.44 (47.5–100)	0.210
CAL	2.27 (1.96–3.42)	2.75 (2.19–3.24)	3.6 (2.9–4.47)	0.002

^a^ Kruskal–Wallis test. ^b^ No. of MT, number of missing teeth; PD, average probing depth; GR, average gingival recession; FD, number of teeth with furcation defect; TM, average level of tooth mobility; FMPS, Full Mouth Plaque Score; FMBS, Full Mouth Bleeding Score; CAL, average clinical attachment level. ^c^ Medians with interquartile range, IQR in parentheses are presented for all variables.

**Table 3:** Predictor of periodontitis in logistic regression (average attachment loss ≥ 4 mm).

Model	Variable	B	SE	*p*	OR (95% CI)
Model 1	Cigarette smoking	1.2	0.7	0.094	3.4 (0.8–14.0)
	Daily nicotine consumption (≥10 cigarettes/heat sticks),	1.4	0.9	0.110	3.9 (0.7–21.1)
	Constant	−3.1	0.8		
Model 2	Cigarette smoking	1.5	0.7	0.027	4.7 (1.2–18.3)
	Constant	−2.3	0.5		

B—logistic coefficient, SE—standard error, OR—odds ratio, *p*—level of significance, CI—confidence interval.

## Data Availability

The data presented in this study are available on public repository of Faculty of Dental Medicine, University of Rijeka, Croatia. “https:/dabar.srce.hr/en/islandora/object/fdmri%3A230” (accessed on 4 January 2024) Špalj S. Okolišni čimbenici i mikrobiološke interakcije u strukturi dentalnog biofilma:istraživački podaci. [Internet]. Fakultet dentalne medicine; 2023, [cited 4 January 2024]. Available from: https://urn.nsk.hr/urn:nbn:hr:271:843444 (accessed on 4 January 2024).

## Appendix A. Questionnaire

Research Questionnaire

Data about the patient:

Respondent’s protocol number:

Gender: □M □F

Age:

Phone number:

Address:

E-mail address:

Questions have only one possible answers unless otherwise indicated.

1. How often do you have dental visits?
  □ every 3 months
  □ every 6 months
  □ every 12 months
  □ more than a year
2. To maintain oral hygiene, I use (multiple answers possible):
  □ toothbrush
  □ dental floss
  □ interdental brushes
  □ tooth paste
  □ mouth rinse
  □ other____________________
3. How many times a day do you brush your teeth?
  □ I do not brush my teeth every day
  □ 1 time
  □ 2 times
  □ 3 times
  □ >4 times
4. I am:
  □ Smoker of classic cigarettes
  □ Smoker of THS (tobacco heating system—IQOS)
  □ Non-smoker
5. How long have you been smoking only classic cigarettes/only IQOS:
  □ 1–2 years
  □ 2–5 years
  □ 5–10 years
  □ >10 years
6. Number of smoked cigarettes/tobacco heat sticks (Heat Stick, HEETS) in 1 day:
  □ <5
  □ 5–10
  □ 10–15
  □ >15
7. Before you started smoking IQOS you:
  □ were a non-smoker
  □ smoked classic cigarettes
  □ smoked e-cigarettes
  □ something else __________________
